# The Effectiveness of Ozone Infiltration on Patient-Reported Outcomes in Low Back Pain: A Systematic Review and Meta-Analysis

**DOI:** 10.3390/life14111406

**Published:** 2024-10-31

**Authors:** Rafael Llombart-Blanco, Gonzalo Mariscal, Violeta Cordón, Carlos Barrios, María Benlloch, Rafael Llombart-Ais

**Affiliations:** 1 Orthopedic Surgery Department, University Clinic of Navarra, 31008 Pamplona, Spain; rllombartb@gmail.com; 2 Institute for Research on Musculoskeletal Disorders, School of Medicine, Valencia Catholic University, 46001 Valencia, Spain; gonzalo.mariscal@mail.ucv.es (G.M.); violetacordon@icloud.com (V.C.); carlos.barrios@ucv.es (C.B.); rllombartivcot@gmail.com (R.L.-A.); 3 Department of Basic Biomedical Sciences, Catholic University of Valencia, 46001 Valencia, Spain; 4 Traumacenter, Casa de Salud Hospital, 46021 Valencia, Spain

**Keywords:** ozone, patient-reported outcomes, low back pain, meta-analysis

## Abstract

Background/Objective: The objective of this systematic review and meta-analysis was to evaluate the current evidence on the relative efficacy of ozone injections for improving patient-reported outcomes (PROMs). Methods: A literature search was conducted in four databases, and PROMs were analyzed. Odds ratios (ORs), mean differences, and standard mean differences with 95% confidence intervals (CI) were calculated. Meta-analysis was performed using Review Manager. Results: Nine studies (total number of participants = 1711) were included. Overall, the ODI (Oswestry Disability Index) showed favorable results for ozone (SMD −0.28, 95% CI −0.51 to −0.06). However, subgroup analysis by follow-up time found no significant differences at 2 weeks or 6 months but significant differences at 1 month. The VAS also favored ozone use overall (SMD −0.12, 95% CI −0.24 −0.01). Subgroup and sensitivity analyses revealed no significant differences between groups. There were also no significant differences in excellent outcomes (OR 0.95, 95% CI 0.54 to 1.67) or poor outcomes (OR 1.25, 95% CI 0.66 to 2.37). Conclusions: Ozone therapy has demonstrated limited benefits over the usual care for lower back pain. While a small advantage emerged for function at one month, the results were inconsistent, and no differences were seen in pain or clinical outcomes.

## 1. Introduction

Low back pain (LBP) is currently the most common condition leading to limited activity and loss of quality of life in young and middle-aged adults [1]. It affects 80% of the population at least once in their lifetime [2], and its estimated prevalence in adults aged 40–80 years old is 11.9% [3,4]. Specific pathologies that contribute to LBP include injuries such as disc herniations, fractures, and strains/sprains, as well as degenerative changes such as disc degeneration and facet joint osteoarthritis [5].

In recent years, interventional procedures have also gained acceptance, such as the infiltration of therapeutic agents into the epidural and periradicular spaces via translaminar or transforaminal routes. Another developed interventional modality is the epidural or periradicular delivery of oxygen–ozone gas mixtures, either as primary treatment or in combination with corticosteroids, for purported short-term pain alleviation [3].

There is ongoing debate regarding the efficacy of ozone therapy for low back pain [2,6,7,8]. Some studies have found potential benefits, such as the study by Andreula et al., in which disc herniation was unresponsive to conservative care [6]. Another study by Bonetti et al. reported that ozone treatment effectively reduced acute and chronic low back pain and sciatica [2]. The combination of ozone with other agents was also investigated. For instance, Gallucci et al. found that intraforaminal/intradiscal injections containing steroids, anesthetics, and ozone were superior to steroids/anesthetics alone for 6-month relief [7]. However, not all evidence has been supportive. Fan et al. found no additional benefit of adding ozone to central epidural steroid injections [8]. The existing evidence on the efficacy of ozone therapy for lower back pain warrants updating. A meta-analysis published in 2019 pooled data from only three studies [3]. Numerous studies evaluating this intervention have been conducted in recent years. A systematic review and meta-analysis incorporating these newer studies is timely as it could help address some of the limitations of prior work. Specifically, a new analysis would enhance the statistical power through the inclusion of a larger body of evidence. It could also expand the scope of outcomes analyzed beyond just 6-month of pain relief, as assessed previously. Evaluating other important patient-centered measures, such as function and quality of life, over varied follow-up durations would provide clinicians with more comprehensive insights. The objective of this systematic review and meta-analysis was to evaluate the efficacy of ozone therapy compared to conventional medical management in improving pain and functional outcomes in adult patients with low back pain.

## 2. Materials and Methods

### 2.1. Eligibility Criteria

**Figure 1:**
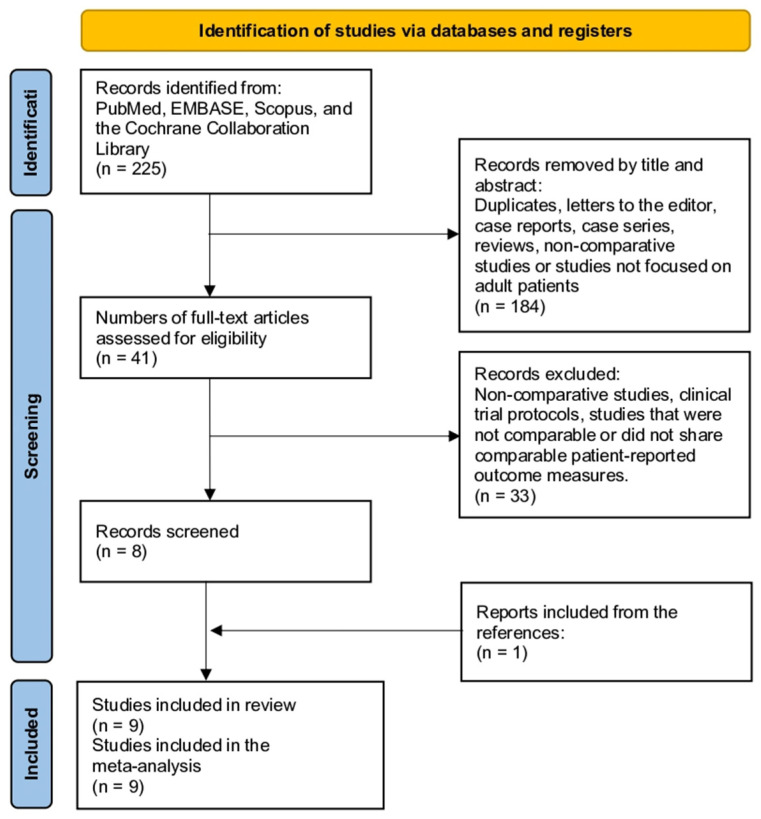
Study selection flow diagram (preferred reporting items for systematic reviews and meta-analyses).

This systematic review and meta-analysis followed a predefined protocol registered with (PROSPERO: CRD42024511949). We conducted the study according to PRISMA guidelines to ensure transparency and reproducibility (Figure 1) [9].

An established PICOS framework was used to systematically identify studies with the following inclusion criteria: adults aged 18 years and older with chronic or recurrent low back pain (P); studies in which participants received ozone therapy comprising intra-discal, paravertebral, or caudal epidural injection of a mixture of oxygen and ozone gas (I); comparisons against standard conservative or interventional care such as steroid injections, placebo injections, or surgery (C); reporting of patient-reported outcome measures as primary outcomes, including pain, functional status, and quality of life (O); and only comparative studies were considered (S). Details of the treatment protocols for the experimental and comparator groups are provided in Supplementary Table S1.

Studies were excluded if they met one or more of the following prespecified criteria: duplicates, case reports and case series, letters to the editor, non-comparative studies, pediatric populations, trial protocols, and studies with incomplete or non-comparable data.

### 2.2. Information Sources

We systematically searched PubMed, EMBASE, SCOPUS, and the Cochrane Library without restrictions on language or publication status. In addition to searching the databases, we also reviewed the reference lists of the included studies.

### 2.3. Search Methods for Identification of Studies

The search strategies combined controlled vocabulary and keyword terms for “ozone therapy” and “low back pain”. An example of the PubMed search strategy is provided in Supplementary File S1. Two independent reviewers screened all retrieved titles and abstracts. Any discrepancies between reviewers were resolved through discussion with a third independent reviewer until a consensus was reached.

### 2.4. Data Extraction and Data Items

Two reviewers minimized the risk of error and bias. Disagreements were resolved by consensus with a third reviewer. The following baseline study characteristics were extracted: study, region, period, follow-up, study design, number of patients, age, female sex, and etiology. Conflicts of interest and funding sources are also recorded. An in-depth description of the experimental and control groups is provided. The primary outcomes that could be compared were the Oswestry Disability Index (ODI) [10], the visual analog scale for back pain (VAS), and the MacNab criteria [11]. Other outcomes that were compared were EQ-5D and SF-36 for quality of life but only “overall health” dimension of both scales.

### 2.5. Assessment of Risk of Bias in Included Studies

For randomized controlled trials, the Cochrane risk-of-bias tool was utilized in duplicate to assess six domains within the Review Manager. Each domain was assigned a low-, high-, or unclear risk rating. These evaluations are presented in Supplementary Figure S1 and Supplementary File S2.

For non-randomized studies, the methodological index for non-randomized studies (MINORS) criteria was employed (Supplementary Table S2) [12].

### 2.6. Assessment of Results

Meta-analyses will be performed using RevMan 5.4 statistical software. Dichotomous outcomes will be analyzed using odds ratios (ORs) with 95% confidence intervals (CIs) to assess differences between groups. Continuous outcomes will be assessed using the mean differences (MDs) or standardized mean differences (SMDs) and associated 95% CIs, as appropriate. Heterogeneity between studies was evaluated using the chi-square test and I^2^ statistics. I^2^ values of 25% represented low heterogeneity, 50% represented moderate heterogeneity, and 75% represented high heterogeneity [13]. A fixed-effects model was used if the heterogeneity was negligible (I^2^ < 75%). When additional outcome data could only be extracted from the figure images, WebPlotDigitizer software version 13.1.4 was used. Guidelines from the Cochrane Handbook will be adhered to when addressing incomplete outcome reporting [14].

### 2.7. Risk of Bias Across the Studies

Publication bias was assessed by visual inspection of funnel plots using Review Manager 5.4, with effect estimates from individual studies plotted against their standard errors. In the absence of publication bias, the plot forms a symmetrical inverted funnel, with effect estimates from smaller studies scattered at the top and increasing precision among larger studies toward the bottom. Asymmetry suggests a possible bias, such as the underreporting of non-significant or negative findings.

### 2.8. Additional Analyses

Subgroup analyses were performed to categorize outcomes based on follow-up time. Sensitivity analyses were performed to assess the robustness of the overall estimates. Specifically, the highest-weighted studies were removed sequentially from the meta-analyses to examine their influence on the pooled effect sizes. Studies were also excluded from the sensitivity analyses based on potential confounding variables. These variables included the location of infiltration (intradiscal, foraminal, or intramuscular), the type of professional performing the procedure, the imaging guidance used (fluoroscopy or CT), and the dose administered (greater or less than 20 mL). A cutoff of 20 mL was chosen, as it divided the studies into two groups, allowing for a balanced representation of the data. The quality of evidence for primary outcomes was assessed using the Grading of Recommendations, Assessment, Development, and Evaluations (GRADE) approach [15].

## 3. Results

### 3.1. Study Selection

The initial search yielded 225 articles. After examining the titles and abstracts, 184 studies were excluded as duplicates, letters, case reports, case series, reviews, or non-comparative studies focusing on pediatric patients. Of the remaining 41 studies, 33 were removed after reading the full text as non-comparative, trial protocol, non-comparable, or did not report comparable patient-reported outcomes. This resulted in eight eligible studies. Further study was performed after screening the references from the included studies. Nine studies were included in the meta-analysis (Figure 1) [2,6,8,16,17,18,19,20,21].

### 3.2. Study Characteristics

Table 1 shows the baseline characteristics of the included studies. This meta-analysis included nine studies with a total of 1711 patients (893 in the ozone group and 818 in the control group). The follow-up period was 1–12 months. The mean age in the ozone group varied from 39.5 and 64.5 years, and in the control group, it ranged from 41.4 to 64.6 years. The etiologies, number of women, conflicts of interest, and funding sources are shown in Table 1. A detailed description of the experimental and control groups is provided in the Supplementary Table S1.

### 3.3. Risk of Bias

Nine studies were randomized, and one was non-randomized. Supplementary Figure S1 and Supplementary File S2 show the quality and risk of bias assessments for the randomized studies. Several studies were judged as having some concerns regarding their methodological quality, with unclear or high risk of bias found in the following domains: allocation concealment, blinding of participants/personnel, and incomplete outcome data. A single non-randomized study was evaluated using the MINORS tool and deemed to be of fair quality (Supplementary Table S2).

### 3.4. Outcomes

**Figure 2:**
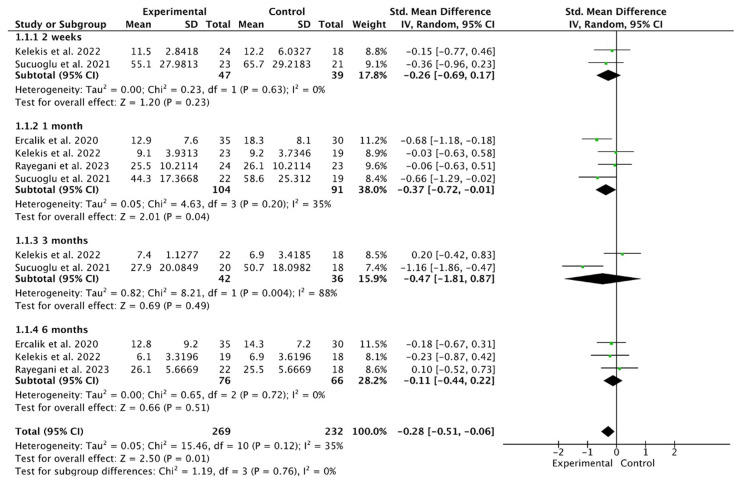
This forest plot depicts the effect of ozone treatment on the Oswestry Disability Index (ODI) scores in patients with LBP at various follow-up intervals []. At the two-week follow-up, the standardized mean difference (SMD) was −0.26, with a 95% confidence interval (CI) ranging from −0.69 to 0.17, indicating no significant differences between the ozone treatment group and the control group. At one month, the SMD was −0.37, and the 95% CI was from −0.72 to −0.01, demonstrating a potentially beneficial effect of the treatment, with a *p*-value of 0.04, suggesting statistical significance. At three months, the SMD was −0.47, accompanied by a 95% CI of −1.81 to 0.87, which again indicated no significant differences between the ozone treatment and control groups. Finally, at the six-month follow-up, the SMD was −0.11, with a 95% CI ranging from −0.44 to 0.22, further showing no significant differences between the intervention and control groups.

Regarding the overall ODI, ozone showed favorable results globally (SMD −0.28, 95% CI −0.51 to −0.06; participants = 501; studies = 11; I^2^ = 35%) (Figure 2).

However, when analyses were conducted according to follow-up time, there were no significant differences at two weeks (SMD −0.26, 95% CI −0.69 to 0.17; participants = 86; studies = 2; I^2^ = 0%). At the monthly follow-up, ozone showed significantly more favorable results (SMD −0.37, 95% CI −0.72, −0.01; participants = 195; studies = 4; I^2^ = 35%). At 3 and 6 months, there were no significant differences: (SMD −0.47, 95% CI −1.81 to 0.87; participants = 78; studies = 2; I^2^ = 88%) and (SMD −0.11, 95% CI −0.44 to 0.22; participants = 142; studies = 3; I^2^ = 0%), respectively. When the study with the highest weight was excluded, no significant differences were observed (SMD −0.24, 95% CI −0.50 to 0.02; participants = 371; studies = 11; I^2^ = 37%). No significant differences were observed at any follow-up time point.

**Figure 3:**
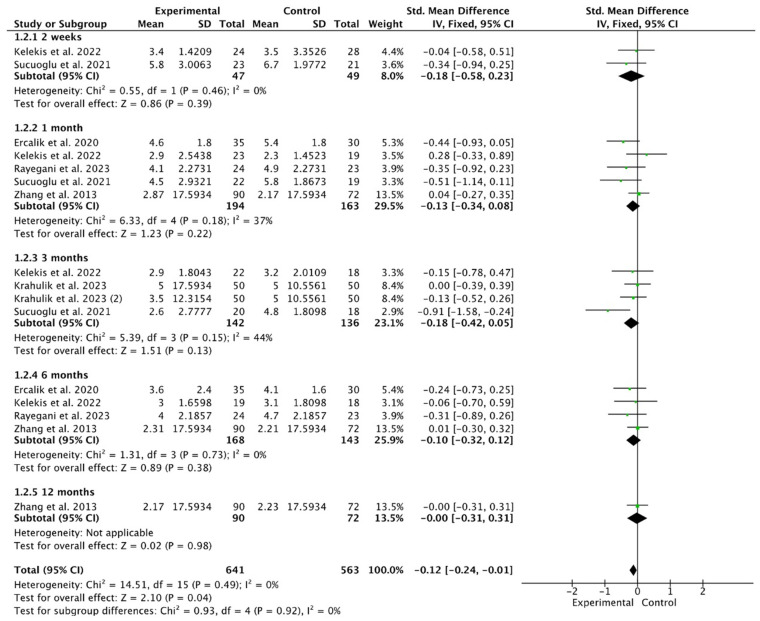
This forest plot illustrates the effect of ozone treatment on the Visual Analog Scale (VAS) scores in patients suffering from low back pain (LBP) across various follow-up periods []. At the two-week mark, the standardized mean difference (SMD) was −0.18, with a 95% confidence interval (CI) ranging from −0.58 to 0.23. At one month, the SMD was −0.13, and the 95% CI was between −0.34 and 0.08. The three-month follow-up revealed an SMD of −0.18, with the 95% CI spanning −0.42 to 0.05. At six months, the SMD decreased to −0.10, with a confidence interval of −0.32 to 0.12. Finally, at twelve months, the SMD was −0.00, and the 95% CI ranged from −0.31 to 0.31. Notably, in all assessed time points, there were no significant differences observed between the ozone treatment group and the control group.

The overall VAS score showed significantly more favorable results in the ozone group (SMD −0.12, 95% CI −0.24, −0.01; participants = 1204; studies = 16; I^2^ = 0%) (Figure 3).

When analyses were conducted according to follow-up time, there were no significant differences at any follow-up time at 2 weeks, 1 month, 3 months, 6 months, and 12 months: (SMD −0.18, 95% CI −0.58 to 0.23; participants = 96; studies = 2; I^2^ = 0%), (SMD −0.13, 95% CI −0.34 to 0.08; participants = 357; studies = 5; I^2^ = 37%), (SMD −0.18, 95% CI −0.42 to 0.05; participants = 278; studies = 4; I^2^ = 44%), (SMD −0.10, 95% CI −0.32 to 0.12; participants = 311; studies = 4; I^2^ = 0%), and (SMD −0.00, 95% CI −0.31 to 0.31; participants = 162; studies = 1; I^2^ = 0%). When the study with the highest weight was excluded, the direction of the results did not change (SMD −0.21, 95% CI −0.36 to −0.07; participants = 718; studies = 16; I^2^ = 0%). There were no significant differences at any follow-up time.

However, there were no differences in the frequency of patients with excellent results (OR 0.95, 95% CI 0.54 to 1.67; participants = 2015; studies = 7; I^2^ = 87%) (Supplementary Figure S2). There were also no differences when divided according to the 1–2 week, 3-month, or 6–12 month follow-up times: (OR 0.49, 95% CI 0.07 to 3.26; participants = 523; studies = 2; I^2^ = 95%), (OR 1.20, 95% CI 0.59 to 2.41; participants = 523; studies = 2; I^2^ = 67%), (OR 1.30, 95% CI 0.82 to 2.08; participants = 969; studies = 3; I^2^ = 61%). When the study with the highest weight was excluded, the direction of the results did not change (OR 0.92, 95% CI 0.46 to 1.84; participants = 1569; studies = 7; I^2^ = 89%).

There were also no significant differences in the number of patients with poor results (OR 1.25, 95% CI 0.66 to 2.37; participants, 2015; studies, 7; I^2^ = 77%) (Supplementary Figure S3). There were also no differences at any 1–2 week, 3-month, or 6–12 month follow-up times: (OR 1.39, 95% CI 0.24 to 7.94; participants = 523; studies = 2; I^2^ = 86%), (OR 1.08, 95% CI 0.30 to 3.90; participants = 523; studies = 2; I^2^ = 66%) and (OR 1.32, 95% CI 0.42 to 4.14; participants = 969; studies = 3; I^2^ = 87%). When the study with the highest weight was excluded, the direction of the results did not change (OR 1.11, 95% CI 0.55 to 2.25; participants = 1569; studies = 7; I^2^ = 71%).

Regarding the quality of life, two reports studied it according to two scales: one used the SF-36 questionnaire and the other used the EQ-5D. Even though these scales encompass multiple dimensions, one dimension, “overall health,” could offer a comparable perspective for our analysis. The results of this forest are shown in Supplementary Figure S4. There were significant differences at one month follow-up time; however, there were no differences at 6 months, according to the rest of the results.

### 3.5. Additional Analyses

Due to the limited number of items on the MacNab scale, these analyses were only performed on the ODI and VAS variables. Table 2 presents the results of this analysis. Both the ODI and the VAS demonstrated similar effects, and doses higher than 20 mL appeared to be more effective, with a standardized mean difference (SMD) of −0.55 (95% CI −0.80 to −0.29) and −0.45 (95% CI −0.70 to −0.20), respectively. There were no significant differences in the injection location between intradiscal and foraminal injections. However, only one study by Sucuoğlu et al. [19] included intramuscular injections, which showed more favorable results both in ODI (SMD −0.69, 95% CI −1.06 to −0.32) and VAS (SMD −0.56, 95% CI −0.93 to −0.20). When considering the professionals who performed the procedure, neurosurgeons yielded more satisfactory results for the ODI (SMD −0.42, 95% CI −0.77 to −0.07), although this could only be compared to one study involving a physical medicine and rehabilitation physician. No significant differences or substantial variations in the results were observed among the other variables analyzed in the subgroup comparisons.

### 3.6. Publication Bias

**Figure 4:**
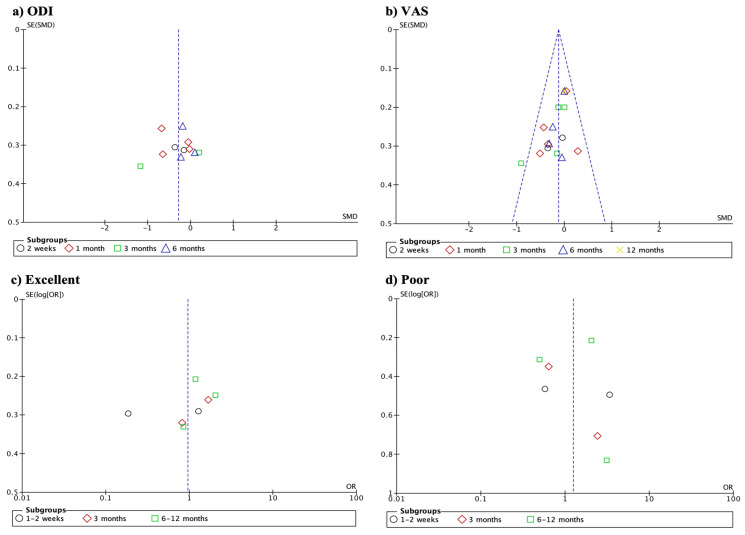
Funnel plot assessing publication bias. Some asymmetry was observed, specifically in the plots for the ODI (**a**), VAS (**b**), the rates of “excellent” (**c**) and “poor” (**d**) outcomes measured by the MacNab scale. Symmetry was observed in VAS outcomes.

Upon visual inspection of funnel plots, some asymmetry was observed, suggesting potential publication bias for certain outcomes. Specifically, asymmetry was present in the plots for the Oswestry Disability Index (ODI) scores and the rates of “excellent” and “poor” outcomes measured by the MacNab scale (Figure 4).

### 3.7. GRADE

The certainty of the included effect sizes according to GRADE is presented in Table 3. The evidence was high for ODI and VAS and moderate for the MacNab scale (excellent and poor results). The variables were weaker in terms of publication bias, differences in the control group, and moderate-to-high risk of bias in some variables.

## 4. Discussion

These results provide limited evidence of the effectiveness of ozone treatment for low back pain. While ozone showed a statistically significant improvement in Oswestry Disability Index (ODI) scores at one month compared to other treatments, indicating less disability, no differences were observed at long-term follow-ups of 3 and 6 months. Similarly, visual analog scale (VAS) pain scores favored ozone globally, but this effect did not persist when analyzing specific follow-up periods of 12 months. No differences were found between the ozone and control groups in terms of the proportion of patients reporting excellent or poor outcomes on the MacNab scale. Some of the original articles included in our meta-analysis considered the oxygen–ozone mixture as a possible treatment for LBP because of the chemical properties of ozone [16,19]. However, no significant differences were observed in this meta-analysis. The reasons given by the original studies are as follows: it is an unstable allotropic form of oxygen weighing 48 kDa, known as O_3_ [6]. It is considered to have analgesic and anti-inflammatory effects [22,23], antiseptic activity [24], and the capacity to cause an oxide reduction called ozonolysis [7]. The O_2_-O_3_ mixture generates reactive oxygen species upon contact with water and fatty acids, initiating an antioxidant cascade that reduces inflammation and modulates pain. This action, combined with the strong diffusion ability of O_2_-O_3_, decreases hernia size, soothes inflamed nerve roots, and provides significant analgesic and anti-inflammatory effects [25]. Thus, ozone could play a role in reverting all pain factors involved in LBP; however, globally, the results are uncertain. Various studies have supported the hypothesis that ozone could contribute to the treatment of LBP by inducing chemonucleolysis in combination with collagenase. Collagenase specifically lyses collagen, one of the main components of protrusions [20]. The results of a study by Wu et al. suggested that the combination of collagenase and ozone to treat lumbar disc herniation improved the treatment success rates [20]. Other authors, such as Andrea et al., consider oxygen-ozone injections as an alternative to enzymatic chemonucleolysis and base this affirmation on the fact that its success rates in their study are similar to those of enzymatic chemonucleolysis. This outcome is important because oxygen–ozone is less invasive due to the following factors: less trauma caused by a narrow needle, no allergic or anaphylactic reactions (0.5% and 0.05%, respectively), no requirement for pre-medication, 2–3 days of bed rest after the treatment compared to 1–2 weeks advised in enzymatic chemonucleolysis, and the possibility of repeating the treatment [6]. In addition, ozone is known for its antiseptic activity, which reduces the risk of post-injection infection. Another way in which ozone can be used to treat LBP is through its anti-inflammatory effects. As reported by Zennaro et al., LBP and sciatica may originate from neurites [26]. The study by Bonetti et al. is based on the fact that episodes of LBP are affected not only by the mechanical compression of the nerve root but also by a non-specific inflammatory reaction to the autoantigens of the mucopolysaccharide matrix, which are located on the surface of the disk and are exposed to the immune system, causing the disk nucleus to migrate beyond the natural barrier of the annulus fibrosus [2]. Therefore, steroids appear to be effective treatment options for LBP. The oxygen-ozone mixture may also help normalize the levels of prostaglandins and cytokines, minimize the levels of reactive oxidant species, increase superoxide dismutase levels, and improve local periganglionic circulation [27,28,29].

Our results are in line with the findings of other authors, such as Hidalgo-Tallón et al., who considered that in practice, there is no level of evidence in the treatment with systemic ozone therapy for chronic pain [30]. Experimental studies have contributed to the understanding of the possible mechanism of action of ozone at the spinal level, yielding encouraging preliminary results that justify further research. One study evaluated the intrathecal administration of low-dose ozone (10–30 μg/mL) in a model of chronic radiculitis, which led to a significant reduction in proinflammatory cytokines, such as TNF-α, IL-6, and IL-1β, and improved mechanical allodynia. This was due to the reduction in the excessive expression of the PDE2A enzyme and NF-κB/p65 factor, which increased the levels of cGMP and cAMP [31]. These findings suggest that radiculitis may be due to the activation of this signaling pathway, which is regulated by ozone. Another study evaluated the possible toxic effects of ozone on spinal cord neurons and demonstrated that 40 μg/mL ozone caused DNA damage and metabolic dysfunction, reducing cell viability, ATP, and NAD+. However, PARP1 inhibition protects these neurons by preventing NAD+ depletion, impacting this enzyme in ozone-mediated toxicity [32]. In contrast, intradiscal administration of ozone in a model of disc degeneration showed protective properties by inhibiting the PI3K/Akt/NF-κB pathway [33]. When analyzing adverse events or complications independently, it was observed that the majority of studies did not find serious events associated with ozone infiltration. Other meta-analyses have also investigated the effects of corticosteroids in the treatment of acute and chronic low back pain. Their results showed a significant reduction in pain in the short term, although it was temporary [34].

When analyzing the overall results, ozone showed statistically significant differences compared to the control group on the VAS pain scale; however, this difference did not reach the minimum clinically important threshold established at 2.6 points for a VAS. The same occurred with the improvement observed in the ODI disability scale at the one-month follow-up, which, although significant, did not exceed the clinically relevant minimum difference of 8.1 points for the ODI [35,36]. In the individual studies, those that showed significant differences between groups, such as the work of Ercalik et al., where the ODI was 12.9 in the ozone group vs. 18.3 in the control group, also did not exceed the MCID [16]. However, Sucuoğlu et al. found an improvement of 44.3 in the ozone group compared to 58.6 in the control group at the one-month follow-up, reaching the minimum clinically significant difference, although transiently at one and three months [19]. Similarly, no individual study has evaluated VAS scores that exceeded the established MCID. Furthermore, the visual review of the results seemed to show a lower magnitude of the differences between the groups as the follow-up time progressed, although this was only a qualitative assessment. Overall, no significant differences in the proportion of patients with excellent outcomes were observed between the ozone group and the control group. This approach, based on dichotomized result analysis (positive vs. negative), provides valuable information, as it is not affected by possible outliers or extremes, as could occur when evaluating continuous scales such as ODI or VAS. Both the ODI and VAS showed the same effect, with doses > 20 mL appearing to be more effective. There were no differences in the injection locations between intradiscal and foraminal injections. In the case of intramuscular injection, only one study by Sucuoğlu et al. showed more satisfactory results both in ODI and VAS; therefore, this finding is limited [19]. Surprisingly, the study by Sucuoğlu et al. consistently showed the best effect for almost all variables and follow-up times [19]. They administered a dose of 30 mL and intra-muscular paravertebral ozone injections. Although the control group did not include corticosteroid injections, it involved a multidisciplinary management approach with a physical therapy (PT) program that included hot packs, ultrasound, TENS, and exercise therapy applied five days a week for three weeks. The medical treatment consisted of oral nonsteroidal anti-inflammatory drugs, opioids, and muscle relaxants administered for 5–14 days [19]. This indicates that this control group may be insufficient compared to using infiltration or surgery as a control group for ozone. Although ozone therapy does not show improvement compared to the control group (mostly corticosteroids), it has demonstrated some benefits. Bhatia reported a 60% improvement in pain and function, although the follow-up time for this outcome was only one month [37]. the same beneficial results, with pain reduction, are described by de Sire et al. [38]. Although no significant long-term differences were found in this meta-analysis, Buric et al. observed a sustained efficacy of intradiscal ozone therapy for up to 10 years. However, this study lacked a control group and did not use validated scales such as the visual analog scale (VAS) or the Oswestry Disability Index (ODI), which are employed in this study to assess patient-reported outcomes [39]. Ozone therapy could be considered an alternative to surgery for patients awaiting surgical intervention, as it has been shown to be a more cost-effective and less hospitalization-intensive approach [40]. However, the published literature has limitations, including short follow-up periods and a lack of comparison with a control group, making it difficult to draw definitive conclusions about its effectiveness.

Bhatia et al. discussed that despite these limitations, ozone therapy may help reduce the use of anti-inflammatory medications with adverse effects for a certain period [37]. Additionally, the type of corticosteroid used in the control group may have influenced the establishment of an appropriate control group. Krahulik et al. (2023) investigated the different types of corticosteroids and found that diprophos, which consists of both a fast-acting and long-lasting depot form of betamethasone, showed better results compared to depomedrone, which only contains methylprednisolone [18]. The lack of conflict of interest in the studies contributes to the reliability and consistency of the results, as it is a quality criterion in meta-analyses according to the AMSTAR-2 scale [41]. Compared to a meta-analysis published in 2019, our study has several strengths. First, we included a greater number of analyses and studies, allowing for a more comprehensive examination of the topic. Furthermore, we provide recommendations for future studies, propose new hypotheses, and offer suggestions on the variables involved in the procedure. A 2010 meta-analysis concluded that ozone therapy is effective and has a good safety profile, with significant clinical improvements in the analyzed scales. However, this meta-analysis included unpublished studies and clinical series. In contrast, our meta-analysis included only comparative studies because of their higher levels of evidence [42]. These contributions enhance the overall value and potential impact of our research and provide valuable insights for further investigations in this field.

### Limitations

Our study has several limitations. First, the sample size was relatively small, which restricted our ability to perform subgroup analyses or meta-regression. Moreover, the follow-up period in many studies included in our analysis was relatively short, with a scarcity of studies providing follow-up data of at least 12 months. Moreover, the selected studies have limited investigation regarding the influence of ozone administration and the experience of the medical practitioner on the effects of oxygen-ozone treatment. In this sense, future studies should focus more on evaluating how the experience of the physician influences outcomes, rather than the specific type of physician performing the procedure. Additionally, the absence of formal statistical tests for publication bias, such as Egger’s and Begg’s tests, in Review Manager 5.4 limits our ability to adequately assess potential publication bias. Furthermore, this analysis did not account for the duration of symptoms, which could potentially influence the treatment outcomes. Notably, studies such as Krahulik et al. employed different control groups, adding complexity to the interpretation of results [17]. There were limited articles available in our analysis that explored the confounding variables. One of these variables is the restricted use of the Oswestry Disability Index (ODI), which has been assessed in nearly half of the studies, recognizing that this could be seen as a drawback. Finally, the specification of the minimum number of studies required in each subgroup analysis to ensure reliable outcomes was not predefined but was instead determined based on the number of studies sharing relevant variables identified after data extraction. This approach may limit the statistical power and reliability of subgroup analyses, as the sample size is contingent upon the availability of data rather than predetermined criteria.

## 5. Conclusions

In conclusion, the application of ozone therapy for the treatment of lumbar pain was subjected to a rigorous analysis within this study. Although the overall Oswestry Disability Index (ODI) indicated a global trend toward favorable outcomes with ozone therapy, the nuanced findings from follow-up intervals present a more complex picture. The lack of significant differences at the majority of follow-up times raises questions about the consistency and durability of the efficacy of ozone in lumbar pain relief. Additional subgroup analyses, including the frequency of excellent and poor results and the effects of varying doses and injection locations, reinforced the main conclusion that ozone therapy has not proven to be consistently effective in managing lumbar pain across the studied population. No significant differences were observed with respect to “excellent” or “poor” results.

Moreover, there is a limited investigation regarding the influence of ozone administration and the experience of the medical practitioner on the effects of oxygen-ozone treatment.

Given these findings, it is important for medical professionals to consider how to counsel patients and apply ozone therapy in clinical practice. Patients should be informed of variable efficacy. Although ozone may play a role when first-line treatments are unsuitable or fail, its use prioritizes options with consistent long-term benefits. Cost-effectiveness should be a factor in planning, given inconsistent results.

## Figures and Tables

**Table 1:** Baseline of all main characteristics of the nine included studies.

Study	Region	Period	Follow-Up (Months)	n Ozone/Control	Age Ozone/Control	Female Ozone/Control	Etiology	Type of Pain	Conflict of Interes (Yes/No)	Funding (Yes/No)
**Andreula et al., 2003** [6]	Italy	1999 to 2001	6	300/300	NR/NR	NR/NR	Lumbar disk herniation	Neurophatic	NR	NR
**Bonetti et al., 2005** [2]	Italy	2001 to 2003	6	156/150	NR/NR	NR/NR	Acute or chronic low back and sciatic nerve pain	Neurophatic	NR	NR
**Ercalik et al., 2020** [16]	Turkey	2018	6	35/30	41.3/49.6	13/16	Lumbar disc herniation	Neurophatic	No	No
**Kelekis et al., 2022** [17]	Greece	2015 to 2018	6	24/23	39.5/41.1	7/9	Lumbar disc herniation	Neurophatic	No	Yes
**Krahulik et al., 2023** [18]	Czech Republic	2019 to 2020	3	50/100	54.2/50.7	27/49	Lumbar disc herniation, foraminal stenosis, lumbar spinal stenosis, spondylolisthesis and postoperative periradicular fibrosis	Neurophatic	No	No
**Rayegani et al., 2023** [8]	Iran	2019 to 2020	6	25/25	64.5/64.6	9/11	Lumbosacral canal stenosis	Neurophatic	No	No
**Sucuoglu et al., 2021** [19]	Turkey	2019 to 2020	1	23/23	45.5/46.9	13/14	Acute lumbar disc herniation	Neurophatic	No	No
**Wu et al., 2009** [20]	China	2003 to 2005	12	108/108	46.5/46.2	50/43	Non-contained lumbar disc herniation	Neurophatic	NR	NR
**Zhang et al., 2013** [21]	China	2005 to 2009	12	90/82	41.5/43.6	41/39	Lumbar disc herniation	Neurophatic	NR	NR

NR: Not reported.

**Table 2:** Sensitivity analysis showing number of participants and subgroup heterogeneity considering confounding variables.

Subgroup	n Participants	Fixed Effect Model (SMD 95% CI)	I^2^ (%)
ODI			
Location of the Infiltration			
**Intradiscal**	422	SMD −0.18, 95% CI −0.37 to 0.01	0%
**Foraminal**	248	SMD −0.03, 95% CI −0.28 to 0.22	0%
**Intramuscular**	123	SMD −0.69, 95% CI −1.06 to −0.32	32%
**Type of professional**			
**Physical medicine and rehabilitation physician**	87	SMD 0.02, 95% CI −0.41 to 0.44	0%
**Neurosurgeon**	130	SMD −0.42, 95% CI −0.77 to −0.07	50%
**Dose administered**			
**>20 mL**	253	SMD −0.55, 95% CI −0.80 to −0.29	33%
**<20 mL**	248	SMD −0.03, 95% CI −0.28 to 0.22	0%
**VAS**			
**Location of the infiltration**			
**Intradiscal**	881	SMD −0.08, 95% CI −0.21 to 0.06	0%
**Foraminal**	951	SMD −0.04, 95% CI −0.17 to 0.09	0%
**Intramuscular**	123	SMD −0.56, 95% CI −0.93 to −0.20	0%
**Type of professional**			
**Physical medicine and rehabilitation physician**	94	SMD −0.33, 95% CI −0.74 to 0.08	0%
**Neurosurgeon**	330	SMD −0.17, 95% CI −0.39 to 0.05	0%
**Dose administered**			
**>20 mL**	253	SMD −0.45, 95% CI −0.70 to −0.20	0%
**<20 mL**	951	SMD −0.04, 95% CI −0.17 to 0.09	0%
**Imaging guidance used**			
**Fluoroscopy**	301	SMD −0.14, 95% CI −0.37 to 0.09	0%
**CT**	686	SMD −0.01, 95% CI −0.16 to 0.14	0%

CI: confidence intervals; CT: computed tomography; SMD: standardized mean differences; ODI: Oswestry Disability Index; VAS: visual analog scale.

**Table 3:** GRADE assessment of the quality of the evidence for primary outcomes and the strength of the recommendations.

Certainty Assessment							№ of Patients		Effect		Certainty	Importance
№ of Studies	Study Design	Risk of Bias	Inconsistency	Indirectness	Imprecision	Publication bias	Clinical	Placebo	Relative (95% CI)	Absolute (95% CI)		
**ODI**												
3	randomized trials	not serious	not serious	serious ^a^	not serious	publication bias strongly suspected	199	172	-	SMD **0.24 lower ** (0.5 lower to 0.02 higher)	⨁⨁⨁⨁ High	CRITICAL
**VAS**												
6	randomized trials	not serious	not serious	serious ^a^	not serious	Not detected	371	347	-	SMD **0.21 lower ** (0.36 lower to 0.07 lower)	⨁⨁⨁⨁ High	CRITICAL
**Excellent**												
2	randomized trials	serious ^b^	not serious	serious ^a^	not serious	publication bias strongly suspected	564/792 (71.2%)	558/777 (71.8%)	**OR 0.92 ** (0.46 to 1.84)	**17 fewer per 1000** (from 179 fewer to 106 more)	⨁⨁⨁◯ Moderate	CRITICAL
**Poor**												
2	randomized trials	serious ^b^	not serious	serious ^a^	not serious	publication bias strongly suspected	74/792 (9.3%)	80/777 (10.3%)	**OR 1.11 ** (0.55 to 2.25)	**10 more per 1000** (from 44 fewer to 102 more)	⨁⨁⨁◯ Moderate	CRITICAL

^a^. Differences in interventions and control group; ^b^. moderate to high risk of bias; CI: confidence interval; OR: odds ratio; ODI: Oswestry Disability Index; VAS: visual analog scale; SMD: standardized mean difference.

## Data Availability

All data generated or analyzed during this study are included in this published article and its Supplementary Information files.

## Supplementary Materials

The following supporting information can be downloaded at: https://www.mdpi.com/article/10.3390/life14111406/s1, Figure S1: Quality and risk of bias assessments for randomized studies were based on the Cochrane Review Manager risk of bias tool; Figure S2: Forest plot showing no significant differences in the frequency of patients with excellent results on the MacNab scale at 1–2 weeks, 3 months and 6–12 months of follow-up; Figure S3: Forest plot showing no significant differences in the frequency of patients with poor results on the MacNab scale at 1–2 weeks, 3 months and 6–12 months of follow-up; Figure S4: Forest plot showing significant differences in quality of life at 1 month of follow-up, but there were no significant differences at 6 months of follow-up; Table S1: Description of the intervention and control group; Table S2: Assessment of the quality of studies through Methodological Index for Non-Randomized Studies (MINORS); File S1: Pubmed search strategy; File S2: Risk of bias judgment.
