# Supplementary material for: The Effectiveness of Ozone Infiltration on Patient-Reported Outcomes in Low Back Pain: A Systematic Review and Meta-Analysis
Source: Life (Basel). 2024 Oct 31;14(11):1406. doi: 10.3390/life14111406 (PMC11595420; doi:10.3390/life14111406)
Supplement: Supplementary file 1 [file life-14-01406-s001.zip › Supplementary Figure S2.pdf]

Study or Subgroup	Experimental Events	Experimental Total	Control Events	Control Total	Weight	Odds Ratio M-H, Random, 95% CI
**1.3.1 1-2 weeks**						
Bonetti et al. 2005	129	156	119	151	14.1%	1.28 [0.73, 2.27]
Wu et al. 2009	33	108	76	108	14.1%	0.19 [0.10, 0.33]
**Subtotal (95% CI)**		**264**		**259**	**28.2%**	**0.49 [0.07, 3.26]**
Total events	162		195			
Heterogeneity: $Tau^2 = 1.79$; $Chi^2 = 21.74$, $df = 1$ ($P < 0.00001$); $I^2 = 95\%$						
Test for overall effect: $Z = 0.74$ ($P = 0.46$)						
**1.3.2 3 months**						
Bonetti et al. 2005	122	156	103	151	14.6%	1.67 [1.00, 2.79]
Wu et al. 2009	80	108	84	108	13.7%	0.82 [0.44, 1.53]
**Subtotal (95% CI)**		**264**		**259**	**28.3%**	**1.20 [0.59, 2.41]**
Total events	202		187			
Heterogeneity: $Tau^2 = 0.17$; $Chi^2 = 3.03$, $df = 1$ ($P = 0.08$); $I^2 = 67\%$						
Test for overall effect: $Z = 0.50$ ($P = 0.62$)						
**1.3.3 6-12 months**						
Andreula et al. 2003	151	211	160	235	15.2%	1.18 [0.79, 1.77]
Bonetti et al. 2005	117	156	90	151	14.7%	2.03 [1.25, 3.31]
Wu et al. 2009	83	108	86	108	13.6%	0.85 [0.44, 1.62]
**Subtotal (95% CI)**		**475**		**494**	**43.5%**	**1.30 [0.82, 2.08]**
Total events	351		336			
Heterogeneity: $Tau^2 = 0.10$; $Chi^2 = 5.10$, $df = 2$ ($P = 0.08$); $I^2 = 61\%$						
Test for overall effect: $Z = 1.12$ ($P = 0.26$)						
**Total (95% CI)**		**1003**		**1012**	**100.0%**	**0.95 [0.54, 1.67]**
Total events	715		718			
Heterogeneity: $Tau^2 = 0.50$; $Chi^2 = 46.29$, $df = 6$ ($P < 0.00001$); $I^2 = 87\%$						
Test for overall effect: $Z = 0.16$ ($P = 0.87$)						
Test for subgroup differences: $Chi^2 = 0.97$, $df = 2$ ($P = 0.61$), $I^2 = 0\%$						

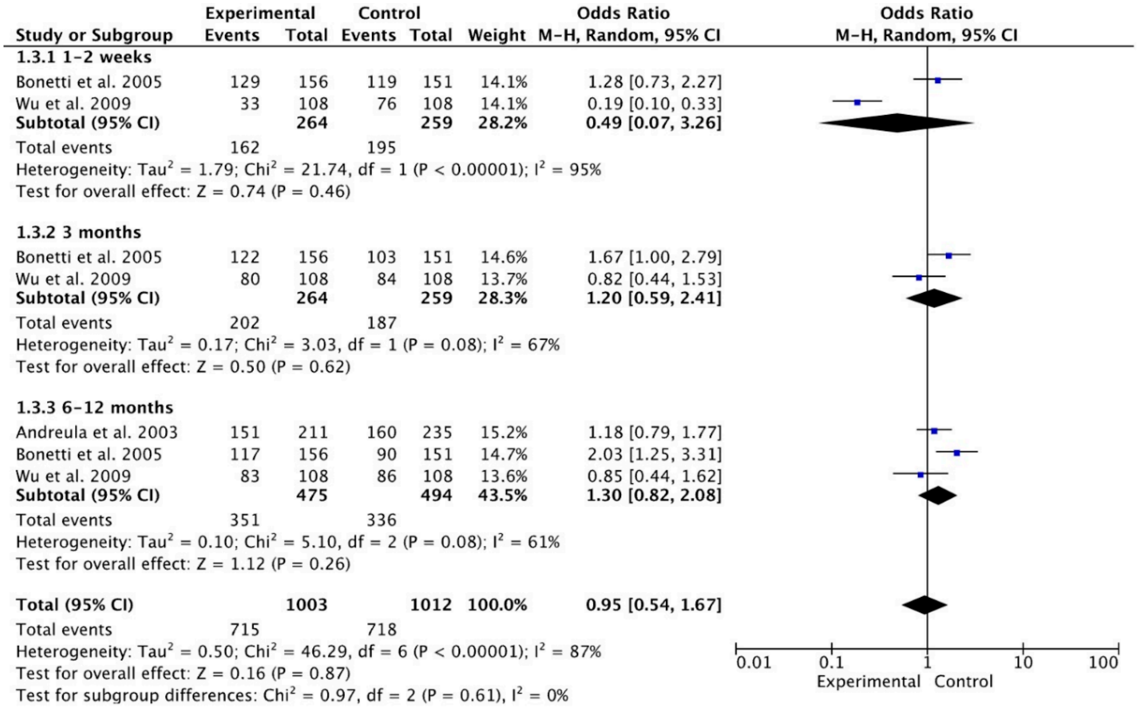

Supplementary Figure S2. Forest plot showing no significant differences in the frequency of patients with excellent results on the MacNab scale at 1-2 weeks, 3 months and 6-12 months of follow-up.
