# Supplementary material for: The Effectiveness of Ozone Infiltration on Patient-Reported Outcomes in Low Back Pain: A Systematic Review and Meta-Analysis
Source: Life (Basel). 2024 Oct 31;14(11):1406. doi: 10.3390/life14111406 (PMC11595420; doi:10.3390/life14111406)
Supplement: Supplementary file 1 [file life-14-01406-s001.zip › Supplementary Figure S3.pdf]

Study or Subgroup	Experimental Events	Experimental Total	Control Events	Control Total	Weight	Odds Ratio M-H, Random, 95% CI
**1.4.1 1-2 weeks**						
Bonetti et al. 2005	8	156	13	151	14.4%	0.57 [0.23, 1.43]
Wu et al. 2009	18	108	6	108	13.9%	3.40 [1.29, 8.94]
**Subtotal (95% CI)**		**264**		**259**	**28.3%**	**1.39 [0.24, 7.94]**
Total events	26		19			
Heterogeneity: $Tau^2 = 1.36$; $Chi^2 = 6.91$, df = 1 (P = 0.009); $I^2 = 86\%$						
Test for overall effect: Z = 0.37 (P = 0.71)						
**1.4.2 3 months**						
Bonetti et al. 2005	16	156	23	151	16.5%	0.64 [0.32, 1.26]
Wu et al. 2009	7	108	3	108	10.5%	2.43 [0.61, 9.64]
**Subtotal (95% CI)**		**264**		**259**	**27.0%**	**1.08 [0.30, 3.90]**
Total events	23		26			
Heterogeneity: $Tau^2 = 0.59$; $Chi^2 = 2.91$, df = 1 (P = 0.09); $I^2 = 66\%$						
Test for overall effect: Z = 0.12 (P = 0.91)						
**1.4.3 6-12 months**						
Andreula et al. 2003	75	211	50	235	18.7%	2.04 [1.34, 3.11]
Bonetti et al. 2005	19	156	33	151	17.1%	0.50 [0.27, 0.92]
Wu et al. 2009	6	108	2	108	8.8%	3.12 [0.61, 15.80]
**Subtotal (95% CI)**		**475**		**494**	**44.7%**	**1.32 [0.42, 4.14]**
Total events	100		85			
Heterogeneity: $Tau^2 = 0.81$; $Chi^2 = 14.89$, df = 2 (P = 0.0006); $I^2 = 87\%$						
Test for overall effect: Z = 0.48 (P = 0.63)						
**Total (95% CI)**		**1003**		**1012**	**100.0%**	**1.25 [0.66, 2.37]**
Total events	149		130			
Heterogeneity: $Tau^2 = 0.53$; $Chi^2 = 26.65$, df = 6 (P = 0.0002); $I^2 = 77\%$						
Test for overall effect: Z = 0.68 (P = 0.50)						
Test for subgroup differences: $Chi^2 = 0.07$, df = 2 (P = 0.96), $I^2 = 0\%$						

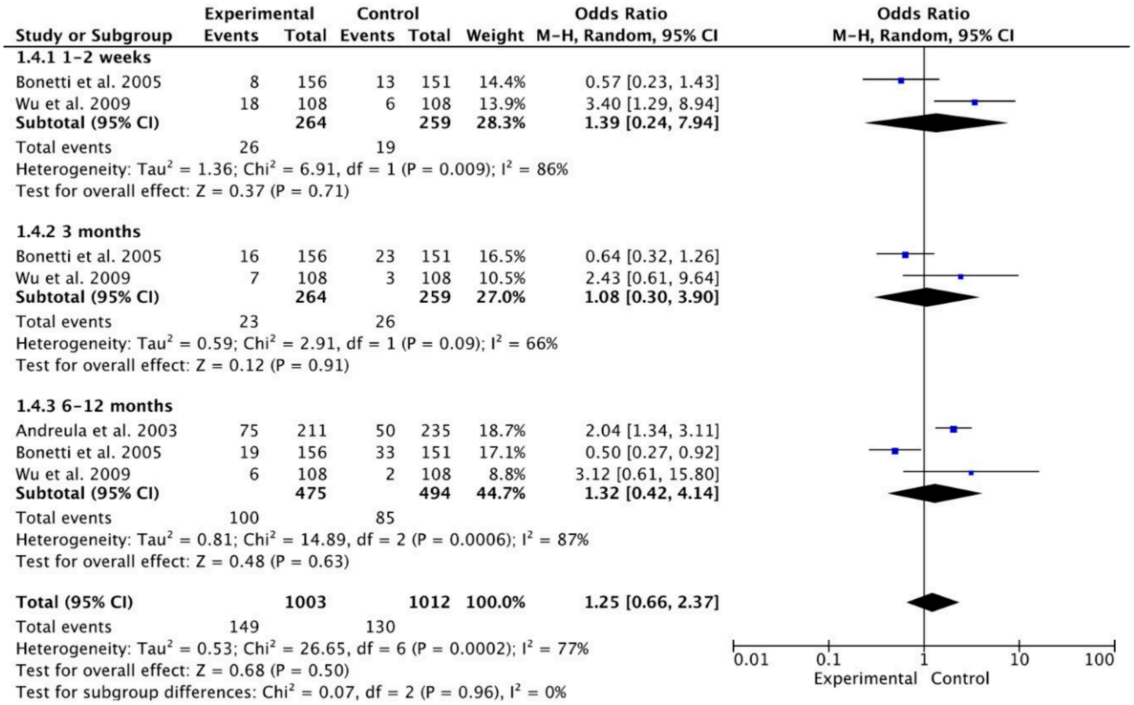

Supplementary Figure S3. Forest plot showing no significant differences in the frequency of patients with poor results on the MacNab scale at 1-2 weeks, 3 months and 6-12 months of follow-up.
