# Supplementary material for: The Effectiveness of Ozone Infiltration on Patient-Reported Outcomes in Low Back Pain: A Systematic Review and Meta-Analysis
Source: Life (Basel). 2024 Oct 31;14(11):1406. doi: 10.3390/life14111406 (PMC11595420; doi:10.3390/life14111406)
Supplement: Supplementary file 1 [file life-14-01406-s001.zip › Supplementary Figure S4.pdf]

Study or Subgroup	Experimental Mean	SD	Total	Control Mean	SD	Total	Weight	Std. mean difference IV, Random, 95% CI
**1.5.1 1 month**								
Ercalik et al. 2020	49.1	15.9	35	40.3	17	30	26.7%	0.53 [0.03 , 1.03]
Kelekis et al. 2022	70.17	9.774543	23	67.58	7.397485	19	23.8%	0.29 [-0.32 , 0.90]
**Subtotal**			**58**			**49**	**50.4%**	**0.43 [0.05 , 0.82]**
Test for overall effect: Z = 2.21 (P = 0.03)								
Heterogeneity: Tau² = 0.00; Chi² = 0.36, df = 1 (P = 0.55); I² = 0%								
**1.5.2 6 months**								
Ercalik et al. 2020	46	16.2	35	54.1	9.4	30	26.6%	-0.59 [-1.09 , -0.09]
Kelekis et al. 2022	72.71	12.038896	19	72.56	11.974479	18	22.9%	0.01 [-0.63 , 0.66]
**Subtotal**			**54**			**48**	**49.6%**	**-0.33 [-0.91 , 0.26]**
Test for overall effect: Z = 1.09 (P = 0.28)								
Heterogeneity: Tau² = 0.10; Chi² = 2.12, df = 1 (P = 0.15); I² = 53%								
**Total**			**112**			**97**	**100.0%**	**0.06 [-0.47 , 0.58]**

Test for overall effect: Z = 0.21 (P = 0.84)
Test for subgroup differences: Chi² = 4.48, df = 1 (P = 0.03), I² = 77.7%
Heterogeneity: Tau² = 0.20; Chi² = 10.56, df = 3 (P = 0.01); I² = 72%

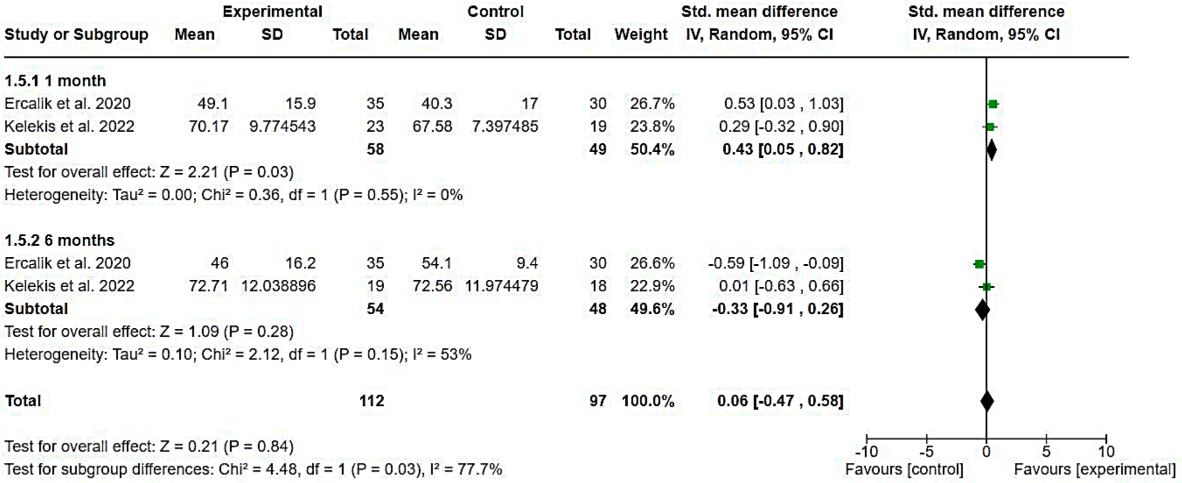

Supplementary Figure S4. Forest plot showing significant differences in quality of life at 1 month of follow-up, but there were no significant differences at 6 months of follow-up.
