# Supplementary material for: The Effectiveness of Ozone Infiltration on Patient-Reported Outcomes in Low Back Pain: A Systematic Review and Meta-Analysis
Source: Life (Basel). 2024 Oct 31;14(11):1406. doi: 10.3390/life14111406 (PMC11595420; doi:10.3390/life14111406)
Supplement: Supplementary file 1 [file life-14-01406-s001.zip › Supplementary File S1.pdf]

Supplementary File S1. Pubmed search strategy.

Search: **ozono AND (lumbar OR "low back pain" OR lumbalgia)**
("ozone"[MeSH Terms] OR "ozone"[All Fields] OR "ozono"[All Fields]) AND ("lumbarised"[All Fields] OR "lumbarization"[All Fields] OR "lumbarized"[All Fields] OR "lumbars"[All Fields] OR "lumbosacral region"[MeSH Terms] OR ("lumbosacral"[All Fields] AND "region"[All Fields]) OR "lumbosacral region"[All Fields] OR "lumbar"[All Fields] OR "low back pain"[All Fields] OR ("low back pain"[MeSH Terms] OR ("low"[All Fields] AND "back"[All Fields] AND "pain"[All Fields]) OR "low back pain"[All Fields] OR "lumbalgia"[All Fields] OR "lumbalgias"[All Fields]))
Translations
ozono: "ozone"[MeSH Terms] OR "ozone"[All Fields] OR "ozono"[All Fields]
lumbar: "lumbarised"[All Fields] OR "lumbarization"[All Fields] OR "lumbarized"[All Fields] OR "lumbars"[All Fields] OR "lumbosacral region"[MeSH Terms] OR ("lumbosacral"[All Fields] AND "region"[All Fields]) OR "lumbosacral region"[All Fields] OR "lumbar"[All Fields]
lumbalgia: "low back pain"[MeSH Terms] OR ("low"[All Fields] AND "back"[All Fields] AND "pain"[All Fields]) OR "low back pain"[All Fields] OR "lumbalgia"[All Fields] OR "lumbalgias"[All Fields]
