# Supplementary material for: The Effectiveness of Ozone Infiltration on Patient-Reported Outcomes in Low Back Pain: A Systematic Review and Meta-Analysis
Source: Life (Basel). 2024 Oct 31;14(11):1406. doi: 10.3390/life14111406 (PMC11595420; doi:10.3390/life14111406)
Supplement: Supplementary file 1 [file life-14-01406-s001.zip › Supplementary File S2.pdf]

Suplementary File S2. Risk of bias judgement.

Bonetti et al. 2005

A) Not reported.
B) Not reported.
C) Not reported.
D) Teams of neuroradiologists (M.B., A.F., B.C.) from two hospitals performed the infiltrations in both groups of patients, and two neurologists (G.D.V., M.G.) blinded to the type of treatment performed the clinical follow-up.
E) Not reported.
F) All kind of outcomes.

Ercalik et al. 2020

A) Randomization was performed using the closed envelope method.
B) Not reported.
C) The physicians and patients that made the evaluations were blinded to the treatment groups.
D) The physicians and patients that made the evaluations were blinded to the treatment groups.
E) The prospective randomized, controlled, doubleblindstudy included 80 patients between 18 and 75years old that had low back/leg pain. While 9 patientsdidn't come for a control examination, 6 patients wereexcluded from the study upon request, and the finalanalyses were performed on 65 patients.
F) All kind of outcomes.

Gallucci et al. 2007

A) Thepatients were randomly assigned to oneof two groups (A and B) by means of arandomization grid.
B) Not reported.
C) Patients were blinded as to whether they had received O2-O3 as part of the treatment.
D) The questionnaire was administered by two individuals (E.S., L.Z.) who were blinded to patient distribution in the two groups.
E) Not reported.
F) All kind of outcomes.

Kelekis et al. 2022

A) Clinical findings of a dermatomal distribution of pain consistent with radiographic findings were also required. Subjects were randomized in a 1:1 ratio to either oxygen-ozone intradiscal injection or microdiscectomy groups. Randomization procedures involved a block design stratified by center using a computerized random number generator allocation sequence. Randomization lists were prepared by the trial's statistician (DH) and the randomization schedule was unknown to treating physicians and allocation of assignments were only provided to them after their randomization request.
B) Randomization procedures involved a block design stratified by center using a computerized random number generator allocation sequence. Randomization lists were prepared by the trial's statistician (DH) and the randomization schedule was unknown to treating physicians and allocation of assignments were only provided to them after their randomization request.
C) It was not possible to blind the interventionalists or the patients to treatments.
D) Outcome assessors were blind to the treatment arm assignment of patients.
E) There were also few patients lost to follow-up, but 7 patients (29%) in the oxygen-ozone group crossed over to microdiscectomy for inadequate symptom relief, almost all (6 patients) within the 3- month follow-up.
F) All kind of outcomes.

Krahulik et al. 2023

A) Not reported.
B) Not reported.
C) Not reported.
D) Not reported.
E) Not reported.
F) All kind of outcomes.

Rayegani et al. 2023

A) In simple randomization with a 1:1 allocation ratio, 50 patients were randomly divided into two groups of 25, selected using a computer-generated list of random numbers.
B) In simple randomization with a 1:1 allocation ratio, 50 patients were randomly divided into two groups of 25, selected using a computer-generated list of random numbers.
C) Participants and assessors were blinded to type of treatment.
D) Participants and assessors were blinded to type of treatment.
E) Figure 1.
F) All kind of outcomes.

Sucuoglu et al. 2021

A) Randomization was done by a physician who did not participate in the recruitment and treatment of the participants. The participants were asked to choose a number between 1 and 10. Those who selected odd numbers were included in the PC group and those who selected an even number were included in the OT group (Fig. 1).
B) Randomization was done by a physician who did not participate in the recruitment and treatment of the participants. The participants were asked to choose a number between 1 and 10. Those who selected odd numbers were included in the PC group and those who selected an even number were included in the OT group (Fig. 1).
C) The physician and patient who performed the assess- 179 ment, administered the POI, planned the PT program 180 and medication, were blinded to the OT doses.
D) The physician and patient who performed the assess- 179 ment, administered the POI, planned the PT program 180 and medication, were blinded to the OT doses.
E) Figure 1.
F) All kind of outcomes.

Wu et al. 2009

A) Patients will be randomly allocated to either minimal invasive group (group A) or conventional discectomy (group B). Randomization will take place in the admitting roomby the research nurse. The researchnurse allocatedthe patients according to the randomnumbers formed by the computer to ensure equal distribution of the randomization treatments.
B) Patients will be randomly allocated to either minimal invasive group (group A) or conventional discectomy (group B). Randomization will take place in the admitting roomby the research nurse. The researchnurse allocatedthe patients according to the randomnumbers formed by the computer to ensure equal distribution of the randomization treatments. The datamanager at the department of biostatistics, who is not involved in the selection and allocation of patients, will prepare coded, sealed envelopes containing the treatment allocation. The treatment were carried out by two specialists who are blind to the study, one is responsible for the minimal invasive procedure, the other is responsible for the conventional open surgery, and they are not allowed to participate the assessment of the results. In the operating room, the surgeon will open the envelope and the allocated treatment is performed. Research nurses are kept blinded for the allocated treatment during the follow-up period of 1 year.
C) Not reported.
D) Research nurses are kept blinded for the allocated treatment during the follow-up period of 1 year. After the treatment, doctor Wang and Wei, who were blinded with the

treatment. Dr. Peng Yang, who is an assistant professor in the department of biostatistics and blinded to the treatment too, carried out the statistical analysis.

E) Not reported.
F) All kind of outcomes.

Zhang et al. 2013

A) All patients were randomly assigned to one of two groups (A and B) by means of a randomization grid.
B) Not reported.
C) Patients were blinded to whether they had received additional betamethasone treatment. Not personnel.
D) Not reported.
E) Not reported.
F) All kind of outcomes.
