# Supplementary material for: The Effectiveness of Ozone Infiltration on Patient-Reported Outcomes in Low Back Pain: A Systematic Review and Meta-Analysis
Source: Life (Basel). 2024 Oct 31;14(11):1406. doi: 10.3390/life14111406 (PMC11595420; doi:10.3390/life14111406)
Supplement: Supplementary file 1 [file life-14-01406-s001.zip › Supplementary Table S1.pdf]

Supplementary Table S1. Description of the intervention and control group

Study	Location of the infiltration	Type of professional	Dose administered	Imaging guidance used	Control group
**Andreula et al. 2003**	Intradiscal and periganglionic	NR	4 mL intradiscal and 8 mL periganglionic	Fluoroscopy	Oxigen-ozone mixture and Depo-Medrone
**Bonetti et al. 2005**	Foraminal.	Neuroradiolo gist	3 mL	NR	Methylprednisolon e acetate and Depomedrone
**Ercalik et al. 2020**	Intradiscal	Neurosurgeon	5 mL	Fluoroscopy	Dexamethasone and bupivacaine
**Kelekis et al. 2022**	Intradiscal	NR	14 mL	Fluoroscopy	Microdiscectomy
**Krahulik et al. 2023**	Periradicular	Neurosurgeon	10 mL	CT	Betamethasone, bupivacaine and methylprednisolon e
**Rayegani et al. 2023**	Foraminal	Physical medicine and rehabilitation physician	10 mL	Ecography	Triamcinolone hexavalent, marcaine and distilled water
**Sucuoglu et al. 2021**	Intramuscular	Physical medicine and rehabilitation physician	30 mL	NR	Hot pack, ultrasound, transcutaneous electrical nerve stimulation and exercise therapy
**Wu et al. 2009**	Foraminal	NR	10 - 15 mL	CT	Discectomy
**Zhang et al. 2013**	Intradiscal	NR	10 mL	CT	Betamethasone

NR: Not reported.
