# Supplementary material for: The Effectiveness of Ozone Infiltration on Patient-Reported Outcomes in Low Back Pain: A Systematic Review and Meta-Analysis
Source: Life (Basel). 2024 Oct 31;14(11):1406. doi: 10.3390/life14111406 (PMC11595420; doi:10.3390/life14111406)
Supplement: Supplementary file 1 [file life-14-01406-s001.zip › Supplementary Table S2 MINORS.pdf]

Supplementary Table S2. Assessment of the quality of studies through Methodological Index for Non-Randomized Studies (MINORS).

Study	Clearly stated aim	Consecutive patients	Prospective collection data	Endpoints	Assessment endpoint	Follow-up period	Loss less than 5%	Study size	Adequate control group	Contemporary group	Baseline control	Statistical analyses	MINORS
**Andreula et al. 2003 [6]**	2	2	2	1	1	2	0	2	0	2	0	1	15
